# Autophagy Impairment Is Associated With Increased Inflammasome Activation and Reversal Reaction Development in Multibacillary Leprosy

**DOI:** 10.3389/fimmu.2018.01223

**Published:** 2018-06-04

**Authors:** Mayara Garcia de Mattos Barbosa, Bruno Jorge de Andrade Silva, Tayná Quintella Assis, Rhana Berto da Silva Prata, Helen Ferreira, Priscila Ribeiro Andrade, Jéssica Araújo da Paixão de Oliveira, Gilberto Marcelo Sperandio da Silva, José Augusto da Costa Nery, Euzenir Nunes Sarno, Roberta Olmo Pinheiro

**Affiliations:** ^1^Leprosy Laboratory, Oswaldo Cruz Institute, Oswaldo Cruz Foundation, Rio de Janeiro, Brazil; ^2^Evandro Chagas National Institute of Infectology, Oswaldo Cruz Foundation, Rio de Janeiro, Brazil

**Keywords:** leprosy, multibacillary patients, reversal reaction, autophagy, inflammasome, *Mycobacterium leprae*

## Abstract

Leprosy reactions are responsible for incapacities in leprosy and represent the major cause of permanent neuropathy. The identification of biomarkers able to identify patients more prone to develop reaction could contribute to adequate clinical management and the prevention of disability. Reversal reaction may occur in unstable borderline patients and also in lepromatous patients. To identify biomarker signature profiles related with the reversal reaction onset, multibacillary patients were recruited and classified accordingly the occurrence or not of reversal reaction during or after multidrugtherapy. Analysis of skin lesion cells at diagnosis of multibacillary leprosy demonstrated that in the group that developed reaction (T1R) in the future there was a downregulation of autophagy associated with the overexpression of *TLR2* and *MLST8*. The autophagy impairment in T1R group was associated with increased expression of *NLRP3*, caspase-1 (p10) and IL-1β production. In addition, analysis of IL-1β production in serum from multibacillary patients demonstrated that patients who developed reversal reaction have significantly increased concentrations of IL-1β at diagnosis, suggesting that the pattern of innate immune responses could predict the reactional episode outcome. *In vitro* analysis demonstrated that the blockade of autophagy with 3-methyladenine (3-MA) in *Mycobacterium leprae*-stimulated human primary monocytes increased the assembly of NLRP3 specks assembly, and it was associated with an increase of IL-1β and IL-6 production. Together, our data suggest an important role for autophagy in multibacillary leprosy patients to avoid exacerbated inflammasome activation and the onset of reversal reaction.

## Introduction

*Mycobacterium leprae* infection results in a chronic disease denominated leprosy (1). The disease presents different clinical forms accordingly the host cellular immune response against mycobacterial antigens and histopathological features (2). *M. leprae* infection can modulate several pathways and modify the microenvironment to favors its survival inside the host cells, including the increase in iron uptake and storage (3, 4), lipids uptake (5, 6), and deactivation of antimicrobial pathways (5, 7).

Our recent study has demonstrated that live *M. leprae* is able to impair the autophagic flux in host cells as an escape immune mechanism (7). Analysis of skin lesion cells demonstrated an upregulation of autophagy genes in paucibacillary tuberculoid and lepromatous patients with reversal reaction when compared with lepromatous patients, which present the progressive form of leprosy (7). Macroautophagy (hereafter termed autophagy) is an evolutionarily conserved mechanism that engulfs targets (e.g., organelles, proteins, and bacteria) through double-membrane vesicles called autophagosomes and directs them for lysosomal degradation [reviewed by Feng et al. (8)].

Basal autophagy is important for the prevention of protein aggregation and the control of reactive oxygen species production (9, 10). Previous studies described autophagy as an important regulatory mechanism controlling unappropriated and potentially deleterious inflammatory responses [reviewed by Harris (11)]. Autophagosomes can sequestrate and degrade inflammasome components including the adaptor ASC, AIM2, NLRP3 (12), and also pro-IL-1β (13). Autophagy inhibition was also described as a potent inflammasome activator, since in the absence of autophagy there is an accumulation of endogenous stimuli (second signal) to inflammasome activation (12–15). Although autophagy machinery is partially impaired in lepromatous patients, the analysis of autophagy genes and proteins expressions during the occurrence of reversal reaction in this group demonstrated that during the reactional episode it was restored (7), maybe mediated by inflammatory mediators, since previous studies have indicated CXCL-10, IL-6, and IFN-γ as biomarkers of reversal reaction (16–19).

Reversal reaction in multibacillary patients is distinguished by the sudden change in the immunological response to mycobacterial antigens and is the leading cause of the leprosy-related morbidity (20–24). The early identification of those episodes is of paramount importance to prevent the neural damage associated with the reactional states. Reversal reaction or Type 1 reaction is an inflammatory exacerbation of skin lesions that may comprise the appearance of new lesions and/or the reactivation of old ones. Reversal reaction may occur across the leprosy clinical spectrum (25). In multibacillary patients, the development of the reversal reaction has been associated with a shift for a Th1 response (26) and although the mechanisms related to the reaction onset are less understood in multibacillary patients, the study of this group permits to identify the role of innate mechanisms related to immunopathogenesis before any previous specific cellular immune response. In addition, due the potential severity of reversal reaction, it is a priority to identify biomarkers of leprosy reaction that may be used to aid the clinicians in patient’s management.

The use of cytokines and chemokines as biomarkers has limitations since they are implicated in various disease states and are not so specific. So, in this study we assessed the pattern of gene expression in skin cells from lepromatous patients who developed or not reversal reaction during or after treatment. We observed that during *M. leprae* infection in monocytes autophagy is important to control inflammasome activation, and that cells from multibacillary patients who did not develop reaction (WR) have increased autophagy when compared with cells from patients who developed reversal reaction (T1R) in the future. The blockade of autophagy in the T1R group cells is accompanied by enhanced NLRP3 inflammasome activation. Therefore, the data presented here suggest that autophagy is important to control of the excessive activation of inflammasome and the possible involvement of NLRP3 inflammasome in the onset of reversal reaction in multibacillary leprosy patients.

## Materials and Methods

### Patients and Clinical Specimens

The participants enrolled in this study, recruited from the Souza Araújo Outpatient Unit (FIOCRUZ), were categorized according to Ridley and Jopling’s classification scale (2). Skin lesion fragments used were obtained *via* 3–6 mm punch, taken from multibacillary leprosy (MB) patients [borderline lepromatous and lepromatous polar (LL)] at diagnosis, prior to treatment, that did not exhibit any signs of leprosy reactions. Blood without anticoagulants was also collected for the obtainment of serum. The patients were monitored for 2 years after the leprosy diagnosis. The patients who developed reversal reaction during this period were included in the T1R group, while the ones that did not develop leprosy reactions were classified as without reaction (WR) (Table 1; Table S1 in Supplementary Material). All the samples used in this study were collected at the multibacillary leprosy diagnosis, no reactional samples were used. The study was endorsed by the Oswaldo Cruz Foundation Human Ethics Committee (CAAE 34239814.7.0000.5248). All the study participants provided informed written consent.

**Table 1:** Baseline characteristics of the multibacillary patients included in the study.

	WR	T1R
**Characteristic**		
Male/female, *n*	5/5	8/4
Age, mean (range)	42.9 (25–65)	44.8 (28–66)
BI, mean (range)	4.19 (1.75–5.85)	3.67 (1–5.50)
LBI, mean (range)	4.84 (3.5–5.85)	4.68 (3.5–5.95)

**Ridley and Jopling clinical form of leprosy, ***n*****		
BL	2	6
LL	8	6

**Leprosy treatment status, ***n*****		
Pretreatment	10	12

*WR, without reaction; T1R, type 1 reaction; BI, bacillary index; LBI, logarithmic bacillary index of skin lesion; BL, borderline lepromatous; LL, lepromatous leprosy*.

### RNA Isolation, Reverse Transcription, and qPCR Analysis

RNA was extracted from the patients skin lesions fragments and blood-derived monocyte cultures by the TRIzol method (Life Technologies, 15596-018) following the manufacturer’s instructions. In order to avoid genomic DNA contamination, the RNA was treated with DNAse (RTS DNase Kit, MO BIO Laboratories); integrity was analyzed *via* 1.2% agarose gel electrophoresis. SuperScript III First-Strand Synthesis System (Life Technologies, 18080-051) was used to perform the reverse transcription. Real-time gene expression was performed using human innate and adaptative immunity (Real-Time Primers, HAIIR-I), and autophagy (Real-Time Primers, HATPL-I) PCR arrays composed of 88 process-related targets and 8 reference genes. The qPCR arrays were performed using the manufacturer-recommended conditions using Power SYBR Green PCR Master Mix (Applied Biosystems, 4367659).

Alternatively, mRNA expression of *NLRP3, IL33, IL18, IL1B*, and *CASP1* was evaluated using TaqMan Fast Universal PCR Master Mix (2×) (Applied Biosystems, 4352042) in a StepOnePlus Real-Time PCR System (Applied Biosystems, MA, USA). All primers were acquired from ThermoFisher Scientific (4331182).

The 2^−ΔCT^ method was used to analyze the gene-expression data, using β-2-microglobulin (*B2M*; Real-Time Primers) as reference gene for the innate and adaptative immunity PCR array, hypoxanthine phosphoribosyltransferase 1 (*HPRT1*; Real-Time Primers) for the autophagy PCR array, and glyceraldehyde-3-phosphate dehydrogenase (*GAPDH*; Hs02758991_g1, Thermo-Fisher Scientific) for the TaqMan assays.

### Pathway Analysis

The RT-qPCR innate and adaptative immunity, and autophagy arrays were used to define the gene-expression profiles of leprosy skin lesions. The disparity in gene-expression profiles between the studied groups were assessed by Linear Model for Series of Arrays (lmFit) and Empirical Bayes Statistics for Differential Expression (ebayes) functions from “limma” (Bioconductor) R package. The differentially expressed genes were identified by log2-fold change ≥ 1.5-fold and moderated by *t*-test *P* value < 0.05 thresholds (5, 7, 27). The Enhanced Heat Map (heatmap.2) function from the “gplots” R package was used to generate the heat maps, displayed in a *z*-scores scaling. Jegga et al. (28) set list of human gene symbols associated to autophagy and lysosomal pathways was used to sub-categorize the differentially regulated autophagy processes-related genes in four functional subgroups (autophagy, autophagy regulators, lysosome, and lysosome regulators).

### Gene Interaction and Enrichment Analysis

The differentially modulated genes in the pathway analyses were evaluated *via* Search Tool for the Retrieval of Interacting Genes/Proteins (STRING) 10.0 database (http://string-db.org/) (29). STRING action and confidence views were used to generate network maps of gene–gene interactions. Gene ontology (GO) and KEGG pathways functional enrichment analysis was generated using the “Enrichment” tool of STRING with false discovery rate and Bonferroni corrections for specific annotations. The *P* < 0.05 threshold was adopted for statistical significance.

### Immunoperoxidase

Frozen skin lesion sections 4-μm thick from LL patients who developed (T1R) or not (WR) episodes of reversal reaction were made in a Leica LM3000 cryostat (Leica, Wetzlar, Germany) and analyzed by the immunoperoxidase technique. The skin sections were fixed with acetone, hydrated with 0.01 M Ca^2+^Mg^2+^-free phosphate-buffered saline (PBS), and the endogenous peroxidase activity was quenched by a 10-min incubation with hydrogen peroxide 0.3% in PBS. Normal horse serum (VECTASTAIN Elite ABC-HRP Kit Mouse IgG, Vector Laboratories, PK-6102) for 30 min at room temperature was used to block unspecific binding sites. The sections were incubated for 1 h at room temperature with 1:50 mouse anti-human LC3 mAb antibody (MBL International, M152-3) diluted in PBS 0.25% Triton X-100 (Sigma-Aldrich, 9002-93-1). After three washes with PBS 0.25% Triton X-100, the slides were incubated for 1 h at room temperature with biotinylated horse anti-mouse IgG (VECTASTAIN Elite ABC-HRP Kit). Next, the sections were washed and incubated with avidin DH-biotinylated horseradish peroxidase (HRP) H complex (VECTASTAIN Elite ABC-HRP Kit) for 40 min for signal amplification. 3-amino-9-ethylcarbazole (AEC Peroxidase HRP Substrate Kit, Vector Laboratories, SK-4200) was used for 10 min at room temperature to develop the reaction. Mayer’s hematoxylin (Dako) was used to counterstain the skin lesion sections. Slides were mounted with aqueous Faramount mounting medium (Dako), and analyzed *via* a Nikon Eclipse E400 microscope with a plan-apochromat 40×/0.65 objective (Nikon Instruments Inc., NY, USA). INFINITYX-32C camera and Infinity Capture software 6.1.0 (Lumenera Corporation, ON, Canada) were used to capture images. LC3-positive area was calculated using Image-Pro Plus 6.0 software (Media Cybernetics, Inc., Rockville, MD, USA) by the ratio between labeled and total tissue areas.

### Skin Lesion Macrophages Isolation

Skin lesion macrophages were isolated as described by Moura et al. (30). Briefly, the dermis was cleaved into small sections and digested overnight at 37°C with 4 mg/mL dispase II (GIBCO, 17105041), and 0.5 mg/mL collagenase type I (GIBCO, 17018029) in RPMI 1640, 10% fetal bovine serum (FBS, GIBCO, 10437028) in a 5% CO_2_ atmosphere. The cell suspension was passed through a 70-µm nylon mesh cell strainer and washed three times with RPMI 1640 by centrifugation at 500 × g for 10 min at 4°C. The cells were resuspended in RPMI 1640 supplemented with 10% FBS, 2 mM l-alanyl-l-glutamine (GlutaMAX I, GIBCO, 35050061), and 100 µg/mL ampicillin (Sigma-Aldrich, A8351), plated at 1 × 10^5^ cells/mL on 15-mm sterile circular coverslips and cultured for 7 days at 37°C in a 5% CO_2_ atmosphere.

### Peripheral Blood Mononuclear Cells (PBMCs) Isolation and Monocyte Cultures

Peripheral blood mononuclear cells from healthy donors were isolated *via* Ficoll-Paque PLUS method (GE Healthcare, 17-1440-03) under endotoxin-free conditions. Cells were ressuspended in RPMI 1640 supplemented with 10% FBS, 2 mM l-alanyl-l-glutamine, 100 U/mL penicillin, and 100 µg/mL streptomycin (Sigma-Aldrich, P4333) and plated at 5 × 10^5^ cells/mL on 15-mm sterile circular coverslips and cultured for 2 h at 37°C in a 5% CO_2_ atmosphere. Alternatively, cells were plated at 1 × 10^6^ cells/mL in 24-well plates for qPCR assays. The supernatant was discarded and coverslips were rinsed with PBS to remove non-adherent cells. The media was replaced and the monocytes were stimulated with armadillo γ-irradiated *M. leprae* at 10 µg/mL (~10:1) in the presence or absence of the following stimuli for 18 h at 37°C in a 5% CO_2_ atmosphere. Autophagy was triggered with 200 ng/mL rapamycin (RP) (Sigma-Aldrich, R0395), 100 µM chloroquine (CQ) (Invitrogen, P36235) was used as an autophagic flux blocker, and 10 µM 3-methyladenine (3-MA) (Sigma-Aldrich, M9281) as an autophagy inhibitor.

### Immunofluorescence Assay

After 7 days of culture, non-adherent cells were removed and the skin lesion macrophages were fixed for 20 min at 4°C using 4% paraformaldehyde (Sigma-Aldrich, 158127). After three washes with PBS 0.05% saponin, cells were blocked for 1 h at room temperature with PBS 10% FBS, 10% normal goat serum (NGS; Sigma-Aldrich, S26-100ML), and 1% bovine serum albumin (BSA; Sigma-Aldrich, 05470-25G). Mouse IgG1 anti-human LC3 antibody (1:50; MBL International, M152-3) was added and incubated overnight at 4°C. Next, the coverslips were washed and 1:500 Alexa Fluor 633 goat anti-mouse IgG1 (Invitrogen, A21126) secondary antibody was added for 2 h at room temperature. DAPI (1:10,000, Molecular Probes) was used to stain the nuclei and the coverslips were mounted in glass slides with Lab Vision PermaFluor Aqueous Mounting Medium (Thermo Scientific, TA-030-FM).

Alternatively, blood-derived monocytes were washed three times with PBS 0.05% saponin and blocked with PBS 10% FBS, 10% NGS, and 1% BSA for 1 h at room temperature. The buffer was then removed and the following primary antibodies were added: rabbit IgG anti-human LC3B (1:100; Novus Biologicals, NB100-2220) and mouse IgG1 anti-human NLRP3 (1:100; abcam, ab17267); and incubated overnight at 4°C. Afterward, the cells were washed and incubated with the secondary antibodies Alexa Fluor 488 goat anti-rabbit IgG (1:500, Invitrogen, A11008) and Alexa Fluor 568 goat anti-mouse IgG1 (1:500; Invitrogen, A21124) for 2 h at room temperature. Finally, nuclei were stained with DAPI. The coverslips were mounted with PermaFluor Aqueous Mounting Medium.

An Axio Observer.Z1 microscope equipped with a Colibri.2 and ApoTome.2 illumination systems (Carl Zeiss, Oberkochen, Germany), the EC Plan-Neofluar 100×/1.30 oil objective, a digital camera AxioCam HRm and its coupled computer equipped with AxioVision Rel. 4.8.2.0 software (Carl Zeiss) were used to image the cells. The Particle Analyzer plugin from ImageJ software was used to assess the numbers of LC3 fluorescent puncta and NLRP3 specks after image thresholding (7). For both analysis, a minimum of 100 cells per sample were scored for each experiment.

### Protein Dialysis and Immunoblotting

After RNA and DNA extraction by the TRIzol method, the protein phases of the skin lesion fragments were obtained *via* protein dialysis as instructed by the manufacturer. 12% polyacrylamide gel electrophoresis was performed with 10 µg protein extracts. After electrophoresis, the resolved proteins were transferred to Hybond-C Extra nitrocellulose membranes (Amersham Biosciences, RPN303E) *via* an electrophoretic transfer system with cold-block (Bio-Rad, CA, USA). Blocking was made with 5% BSA (Sigma-Aldrich, A2153) in PBS 0.1% Tween-20 for 1 h at room temperature. Next, the primary antibodies rabbit anti-human Caspase-1 p10 (1:200, Santa Cruz Biotechnology, sc-515) and mouse IgG1 anti-human GAPDH (1:500, Santa Cruz Biotechnology, sc-47724) were incubated sequentially in the membranes overnight at 4°C. After washing, appropriate HRP-conjugated secondary antibodies goat anti-mouse IgG-HRP (1:2,000; DakoCytomation, P0447) or goat anti-rabbit IgG-HRP (1:2,000; DakoCytomation, P0448) were added for 1 h at room temperature. Chemoluminescent substrate Western Blotting Luminol reagent (Santa Cruz Biotechnology, sc-2048) was added to detect immuno-reactive band. Blottings were revealed using medical X-ray film (Carestream Kodak X-Omat LS film, Amersham Biosciences, F1149). Densitometric analysis was performed using Adobe Photoshop CC software version 14.2.1.x64 (Adobe Systems Incorporated, USA).

### ELISA

To determine the concentration of IL-1β in the patient’s serum, the Human IL-1 beta ELISA Ready-SET-Go! Kit (Affymetrix, eBioscience, 88-7261-77) was used according to the manufacturer’s directions. Alternativelly, IL-1β, IL-6, and TNF concentrations were evaluated in the monocyte culture supernatants accordingly the instructions of the fabricant (Affymetrix, eBioscience, respectively 88-7261-77, 88-7066-77, 88-7346-77).

### Statistical Analysis

Statistical significance was calculated by Mann–Whitney test or Kruskal–Wallis with Dunn’s multiple comparison post-test *via* GraphPad Prism 5.00.288 software (GraphPad, La Jolla, CA, USA). A *p* < 0.05 was deemed statistically significant.

## Results

### Innate Immunity Is Differentially Regulated in WR versus T1R Patients

To identify possible markers of future development of reversal reaction in multibacillary patients, isolated mRNAs of skin lesions obtained at diagnosis were evaluated by RT-qPCR array for innate and adaptative immunity genes.

**Figure 1:**
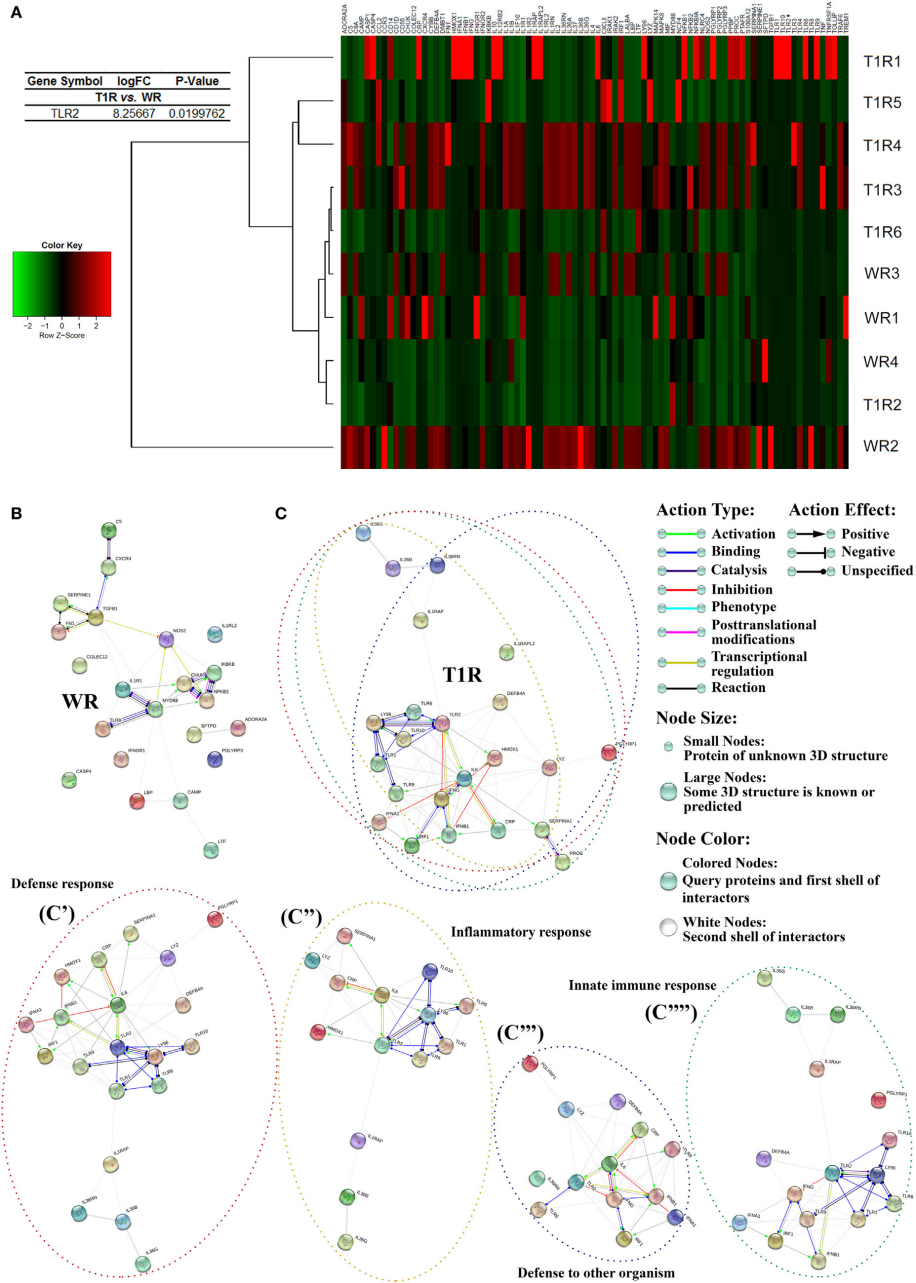
Gene-expression profile of leprosy lesions showed a modulation of innate and adaptative immunity-associated genes between multibacillary patients who developed (T1R) or not (WR) reversal reactional episodes in the future. Purified mRNAs from skin lesions of multibacillary patients who developed or not reversal reaction episodes were analyzed by RT-qPCR innate and adaptative immunity array. The expression fold values of the significantly upregulated genes in WR and T1R lesions were tabulated (full data are available in Table S2 in Supplementary Material). The threshold for statistical significance was *p* < 0.05. **(A)** Heat map showing analysis of differential expression of innate and adaptative immunity-related genes in leprosy patients. Each row represents one donor. Asterisks indicate genes with differential expression. Heat map data are representative of four WR and six T1R samples. **(B,C)** Innate and adaptative immunity gene interaction networks in WR and T1R skin lesions. Genes with a differential expression in leprosy lesions according to autophagy PCR array analysis were visualized by STRING. The action network view. In this view, colored lines and arrow styles between genes indicate the various types of interactions. Network nodes represent genes. Edges represent gene–gene associations. **(C)** Interactions in genes annotated to defense response (C′), inflammatory response (C″), defense to other organism (C′″), and innate immune response (C″″) ontology terms in T1R group patients are shown. Interaction maps are representative of four WR and six T1R samples.

The gene-expression profiles of multibacillary patients showed a differential regulation of innate and adaptative immunity between patients who developed (T1R) or not (WR) reversal reaction episodes in the future. Patients of the group T1R showed a significative increase in *TLR2* expression (Figure 1A; Table S2 in Supplementary Material), as well as a predominance of genes related to pro-inflammatory responses, as *CRP, IL36B, IL36G, IL36RN, IL6, IFNG*, and *LY96*, type 1 interferon pathway, as *IRF1, IFN1, IFNA1, IFNB1*, TLR activation, as *TLR1, TLR6, TLR9, TLR10*, inflammasome activation, as *IL1RAP* and *IL1RAPL2*, and antimicrobial responses, as *DEFB4A* and *LYZ* (Figure 1A; Table S2 in Supplementary Material).

Conversely, WR patients lesions present an increased expression of the genes inhibitors of NFκB, *IKBKB*, and *CHUK*, the immunoregulator *TGFB1*, the antimicrobial peptide codifier *CAMP, TLR8*, extracellular matrix protein, *FN1, NFKB2*, the scavenger receptor of oxidatively modified low-density lipoprotein *COLEC12*, the inflammatory caspase *CASP4*, and the cytokine receptors *IL1RL2* and *IFNGR1* (Figure 1A; Table S2 in Supplementary Material).

The genes differentially expressed between the two groups of multibacillary patients were submitted to gene–gene interactions and enrichment analysis using the STRING database. Network maps of upregulated genes in leprosy skin lesions showed more interactions between innate and adaptative immunity-associated genes in T1R than WR patients (Figures 1B,C; Figure S1 in Supplementary Material). GO enrichment analysis of canonical pathways showed that T1R lesions were predominantly enriched for innate immunity-associated GO terms such as defense response, inflammatory response, defense to other organism, innate immune response, and so on, as compared to WR lesions (Figure 1C).

There were not significantly changes in TLR2 gene and protein expression in *M. leprae*-stimulated primary monocytes from T1R and WR patients (data not shown), suggesting the existence of a specific immune response in skin. Taken together, those data indicate a predominance of TLR and inflammasome activation in skin cells, as well as of pro-inflammatory responses in patients who developed reversal reactional episodes in the future rather than the WR group.

### Autophagy Is Differentially Regulated in WR versus T1R Patients

The prior results indicated that the innate immune response gene activation was upregulated in skin lesions of multibacillary patients who developed reversal reactional episodes (Figure 1). To further identify the host pathways involved in leprosy immune response, we analyzed the transcriptional regulation of the autophagic pathway, an innate mechanism recently described to be implicated in leprosy polarization (7). In this order, the ATG (autophagy-related) gene-expression profile of multibacillary skin lesions mRNAs was analyzed by RT-qPCR using a human autophagy pathway PCR array.

**Figure 2:**
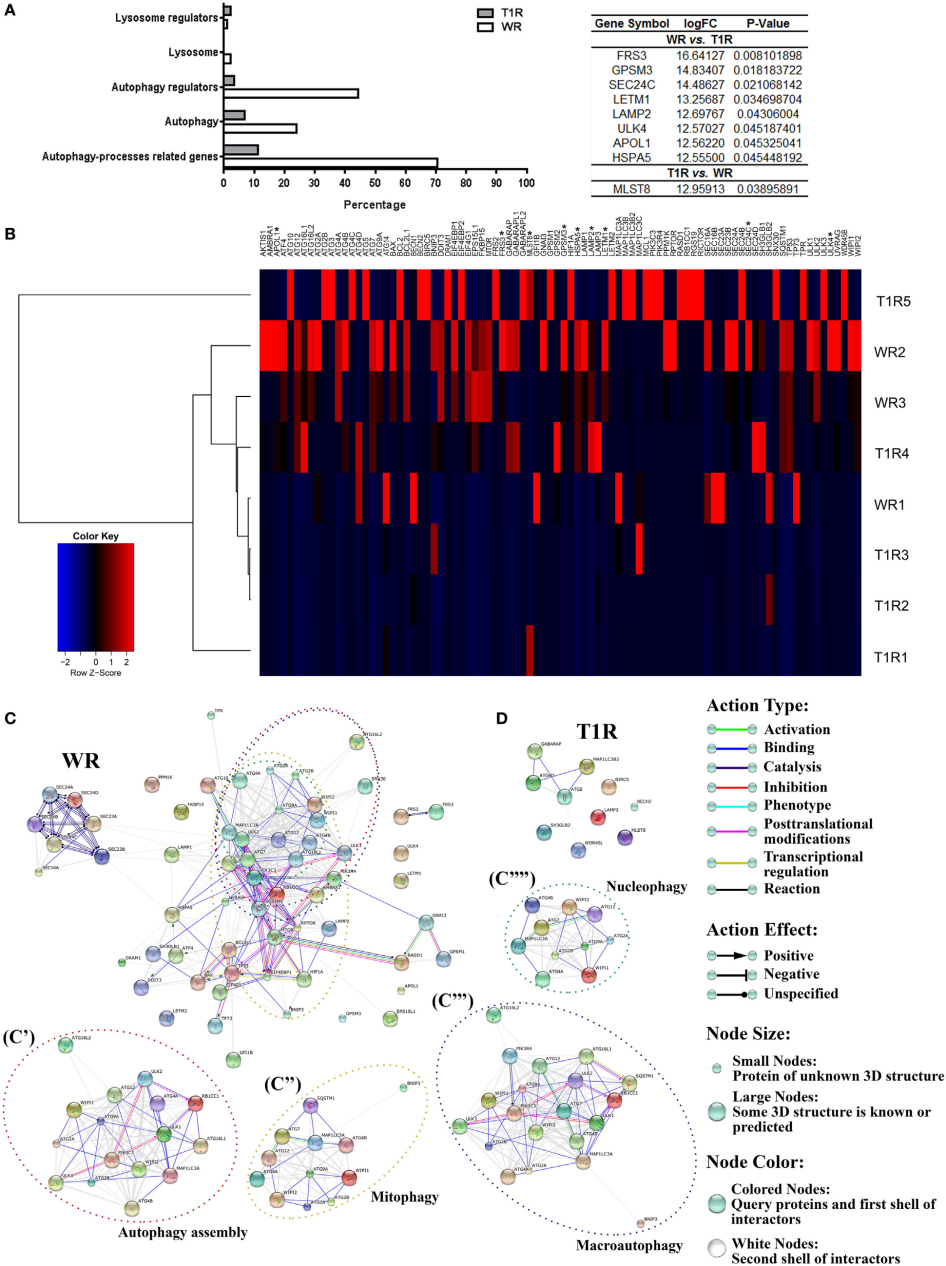
Gene-expression profile of leprosy lesions showed a modulation of autophagy-associated genes between multibacillary patients who developed (T1R) or not (WR) reversal reactional episodes in the future. Purified mRNAs from skin lesions of multibacillary patients who developed (T1R) or not (WR) reversal reactional episodes were analyzed by RT-qPCR autophagy array. **(A)** Differentially expressed autophagy processes-related genes were sub-categorized. The expression fold values of the significantly upregulated genes in WR and T1R lesions were tabulated (full data are available in Table S3 in Supplementary Material). The threshold for statistical significance was *p* < 0.05. **(B)** Heat map showing analysis of differential expression of autophagy processes-related genes in leprosy patients. Each row represents one donor. Asterisks indicate genes with differential expression. Heat map data are representative of three WR and five T1R samples. **(C,D)** Autophagy gene interaction network in WR and T1R skin lesions. Genes with a differential expression in leprosy lesions according to autophagy PCR array analysis were visualized by STRING. The action network view. In this view, colored lines and arrow styles between genes indicate the various types of interactions. Network nodes represent genes. Edges represent gene–gene associations. Interactions among autophagy processes-related genes were more evident in WR than T1R patients. **(C)** Interactions in genes annotated to autophagy assembly (C′), mitophagy (C″), macroautophagy (C′″), and nucleophagy (C″″) ontology terms in WR group patients are shown. Interaction maps are representative of three WR and five T1R samples.

Surprisingly, multibacillary patients that did not develop reversal reactional episodes (WR, 70% of genes) presented a strong upregulation of several autophagy processes-related genes versus those who developed (T1R, 11%) by fold-change analysis. Upregulated genes in multibacillary lesions are involved in regulation of autophagy (44% of WR versus 3% of T1R), autophagosome formation (24% of WR genes versus 6% of T1R genes), lysosomal function or pathways (2% of WR versus 0% of T1R), and regulation of the lysosome (1% of WR versus 2% of T1R) by using Jegga et al. (28) functional autophagy-lysosomal gene classification (Figure 2A; Table S3 in Supplementary Material).

Furthermore, WR lesions displayed a significantly higher expression of eight genes, most involved in autophagy regulation, when compared to just one in T1R lesions. WR lesions presented a highly significant expression of *FRS3, GPSM3, SEC24C, LETM1, LAMP2, ULK4, APOL1*, and *HSPA5* (Figures 2A,B; Table S3 in Supplementary Material). Many other genes of the core autophagic machinery were also upregulated in the WR skin lesions, as members of the Atg1/ULK complex (*ULK1* and *3*), the first complex to regulate autophagosome assembly, Atg9 and its cycling system (*ATG2A/B, ATG9A*, and *WIPI1/2*), which has a role in supplying membranes for phagophore expansion, the PIK3 complex (*PIK3C3* and *PIK3R4*) that participates in the vesicle nucleation stage and promotes the recruitment of PI3P-binding proteins to the site of phagophore biogenesis, the Atg8 (*ATG4A, ATG7*, and *MAP1LC3A*) and Atg12 conjugation systems (*ATG7, ATG10, ATG12, ATG16L1*, and *L2*) two ubiquitin-like conjugation systems involved in vesicle expansion, and the lysosomal components (*LAMP1* and *2*) required for autophagosome-lysosome fusion step (Figure 2B; Table S3 in Supplementary Material) [reviewed by Feng et al. (8)].

On the other hand, in T1R lesions a significant expression of the MTOR complex gene *MLST8* was found. Fold change analysis showed an increased expression of a subset of genes also implicated in autophagy activation, such as *WDR45B*, a component of Atg9 and its cycling system, *LAMP3*, a lysosomal system constituent, *ATG4D, GABARAP, MAP1LC3B2*, members of the Atg8 conjugation system, *ATG5*, a component of the Atg12 conjugation system, *BIRC5*, a member of the inhibitors of apoptosis gene family, *SH3GLB2*, a component of the phagophore membrane curvature complex, and *BECN2*, a mammal-specific homolog of the PIK3 complex gene *BECN1* (Figure 2B; Table S3 in Supplementary Material) [reviewed by Feng et al. (8)].

Gene–gene interactions and enrichment analysis were made *via* the STRING database in the differentially regulated genes in multibacillary skin lesions. The superexpressed gene network maps of leprosy lesions displayed an increased number of interactions among autophagy-associated genes in WR when compared to T1R patients (Figures 2C,D; Figure S2 in Supplementary Material). GO enrichment analysis of canonical pathways showed that WR lesions were predominantly enriched for autophagy-associated GO terms such as autophagy assembly, mitophagy, macroautophagy, nucleophagy, and so on, as compared to T1R lesions (Figures 2C,D).

**Figure 3:**
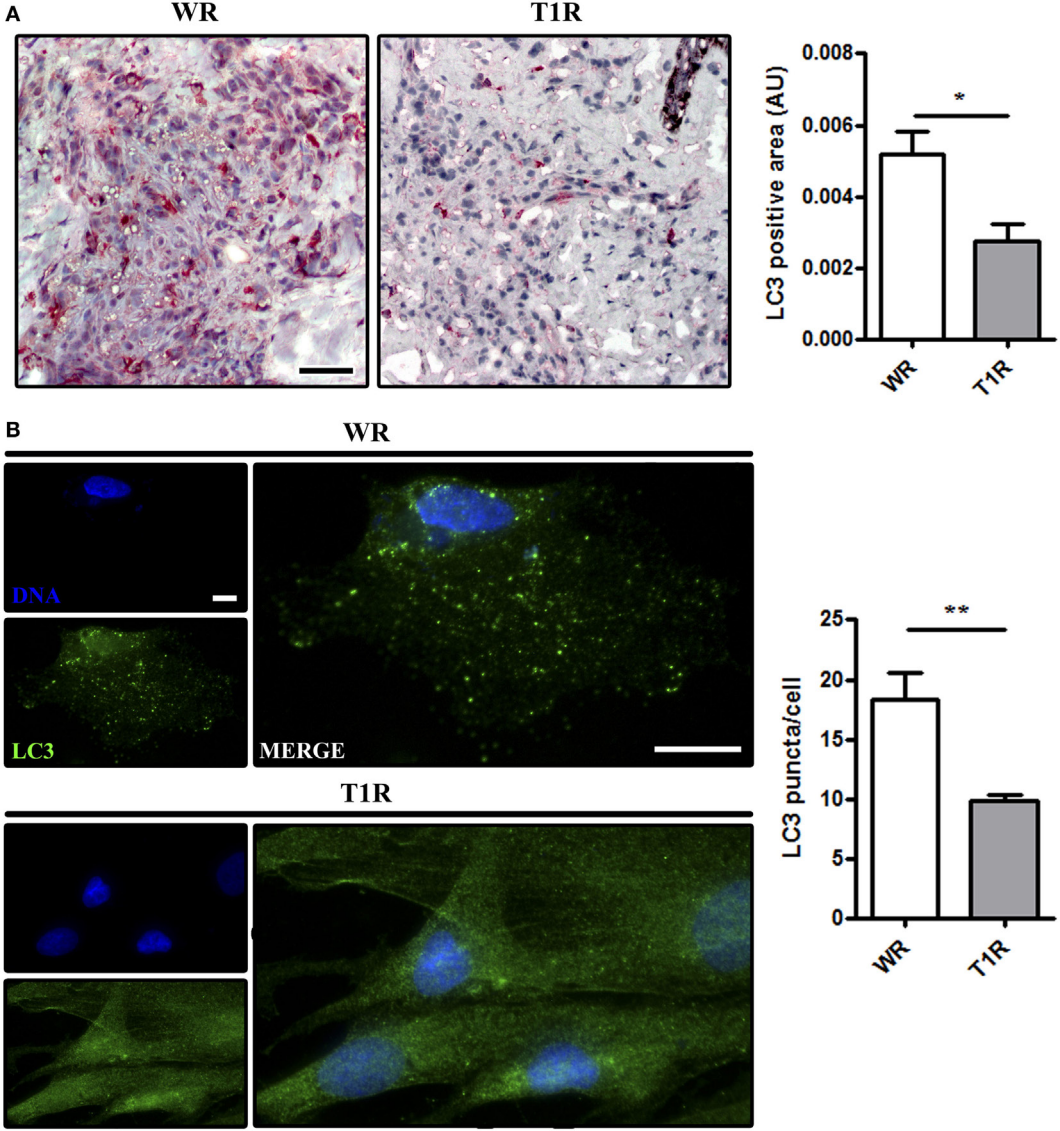
Increase of autophagy levels in skin lesions of multibacillary patients that did not develop reversal reactional episodes (WR). Skin lesion samples were obtained from multibacillary patients who developed (T1R) or not (WR) reversal reactional episodes and analyzed as indicated. **(A,B)** Increased LC3 expression in skin lesion cells of WR patients. **(A)** Immunohistochemical (IHC) analysis of endogenous LC3. Representative micrographs from WR (*n* = 3) and T1R (*n* = 4) patients are shown. IHC images were quantified and data are expressed as arbitrary units (AU). Bars represent the mean values ± SEM. *p < 0.05. Scale bar: 50 µm. **(B)** Macrophages were isolated from skin lesions of multibacillary patients who developed (T1R) or not (WR) episodes of reversal reaction in the future, and cultured for 18 h. Cells were fixed and stained with the anti-LC3 antibody (green) and DAPI (blue). Macrophages of WR skin lesions showed enhanced LC3 puncta formation as compared to T1R macrophages. Immunofluorescence images were quantified and bars represent the mean values of the number of LC3 puncta per cell ± SEM (WR, *n* = 3; T1R, *n* = 3) (**p < 0.01). Scale bar: 20 µm.

PCR array data analysis supplied correlative results regarding increased autophagy in WR as opposed to T1R lesions (Figure 2). Thus, to confirm that there is increased autophagy in multibacillary patients that not undergo reversal reactional episodes (WR), the protein expression of LC3 in WR and T1R lesions was measured. Immunohistochemistry analysis showed a higher presence of endogenous LC3 in non-reactional multibacillary patients (WR) when compared to those who developed reversal reaction lesions (T1R) in the future (Figure 3A). Additionally, skin lesion-derived WR macrophages exhibited higher autophagic puncta formation than macrophages of T1R patients through immunofluorescence staining of the autophagy marker LC3 (Figure 3B). Taken together, our results indicate that the blockade of autophagy may be associated with the occurrence of reversal reaction episodes in multibacillary patients.

### Inflammasome NLRP3-IL-1β Pathway Is Differentially Regulated in WR versus T1R Patients

Since our previous data showed increased expression of IL-1 receptor accessory proteins and a blockage of the autophagic pathway in the multibacillary patients from the group T1R, and several studies report that autophagy blockade can potentialize the inflammasome activation (7, 12–15), we evaluated the expression of the NLRP3 pathway genes, *NLRP3, IL1B, IL18, IL33*, and *CASP1*, in the skin lesion cells by RT-qPCR.

**Figure 4:**
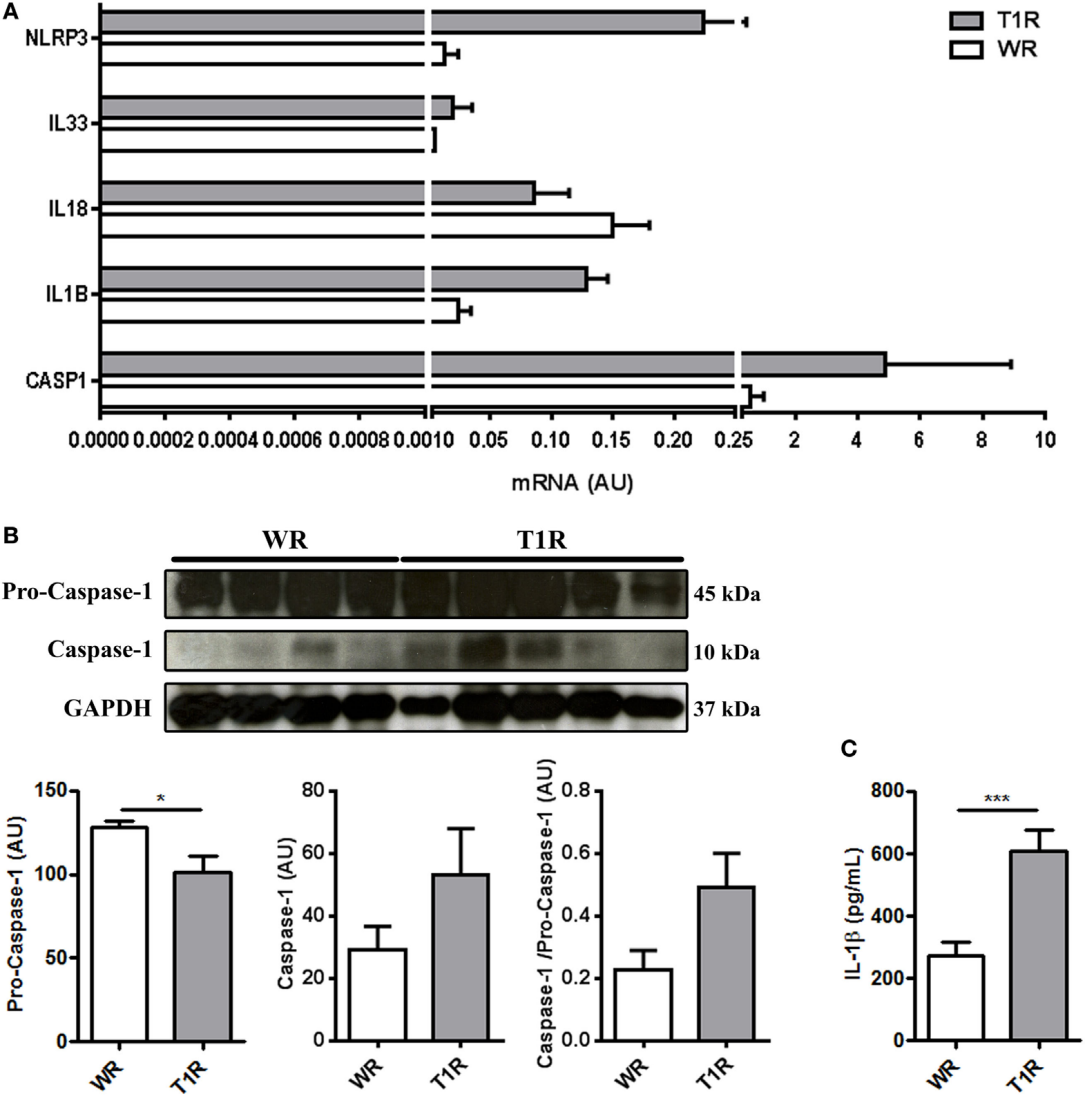
Leprosy lesions present a modulation of inflammasome-associated genes, the subunits of caspase-1 protein, and IL-1 β secretion between multibacillary patients who developed (T1R) or not (WR) reversal reactional episodes in the future. **(A,B)** Skin lesion samples were obtained from multibacillary patients who developed (T1R) or not (WR) reversal reactional episodes and analyzed as indicated. **(A)** Purified mRNAs from skin lesions of multibacillary patients who developed or not T1R episodes were analyzed by RT-qPCR for *NLRP3, IL33, IL18, IL1B*, and *CASP1*. Bars represent the mean values ± SEM of three patients of each group. **(B)** Increased activity of caspase-1 in T1R patients skin lesion. Protein contents from leprosy lesions were analyzed by immunoblotting with anti-caspase-1. Glyceraldehyde-3-phosphate dehydrogenase (GAPDH) was used to verify protein amount loading. Blots are shown (WR, *n* = 4; T1R, *n* = 5). Densitometric analysis of the blots was performed and the caspase-1 (p10 subunit) and pro-caspase-1 (p45 subunit)/GAPDH ratios are expressed as arbitrary units (AU). Data are presented as mean ± SEM. *p < 0.05. **(C)** The IL-1β levels were assessed in the sera of multibacillary patients who developed (T1R) or not (WR) reversal reaction episodes by ELISA. Bars represent the mean values ± SEM (WR, *n* = 10; T1R, *n* = 12). ***p < 0.001.

The T1R samples presented an increased expression of *NLRP3, IL1B*, and *CASP1* (Figure 4A).

Inflammasomes catalyze pro-caspase-1 in caspase-1, an enzyme that in turn proteolytically activates IL-1β [reviewed by Harris (11)].

The increase in the gene expression of the T1R samples was accompanied by increased caspase-1 (p10) and ratio caspase-1-pro-caspase-1 (p10/p45), indicating increased caspase-1 activation and activity in those samples (Figure 4B). On the other hand, samples of the WR group showed accumulation of pro-caspase-1 (p45) confirming our hypothesis (Figure 4B). We also observed increased IL-1β amounts in the sera of patients who developed reversal reactional episodes in the future (Figure 4C).

Taken together, our data indicate increased autophagy in multibacillary patients that did not develop reversal reaction episodes (WR), with accumulation of pro-caspase-1 in the tissue. On the other hand, multibacillary patients who developed reversal reactional episodes (T1R) in the future presented a blockage of autophagy and increased inflammasome activation and consequent IL-1β secretion already at diagnosis time point, 2–20 months before the reactional episode occurrence.

### Autophagy Regulates Inflammasome Activation in *M. leprae*-Stimulated Primary Human Monocytes

To determine whether autophagy affected inflammasome activation during *M. leprae* stimulation, we examined the effect of autophagy activation or blockade on NLRP3 activation in human blood-derived monocytes from healthy donors stimulated with γ-irradiated *M. leprae*. Upon inflammasome activation, NLRP3 recruits the adapter protein ASC and assembles large protein scaffold complexes, which are termed “specks,” which causes caspase-1 activation, resulting in the maturation of IL-1β. Hence, due to the significantly large size of these structures, NLRP3/ASC specks can be effortlessly visualized by fluorescence microscopy as a simple upstream readout for inflammasome activation (31).

**Figure 5:**
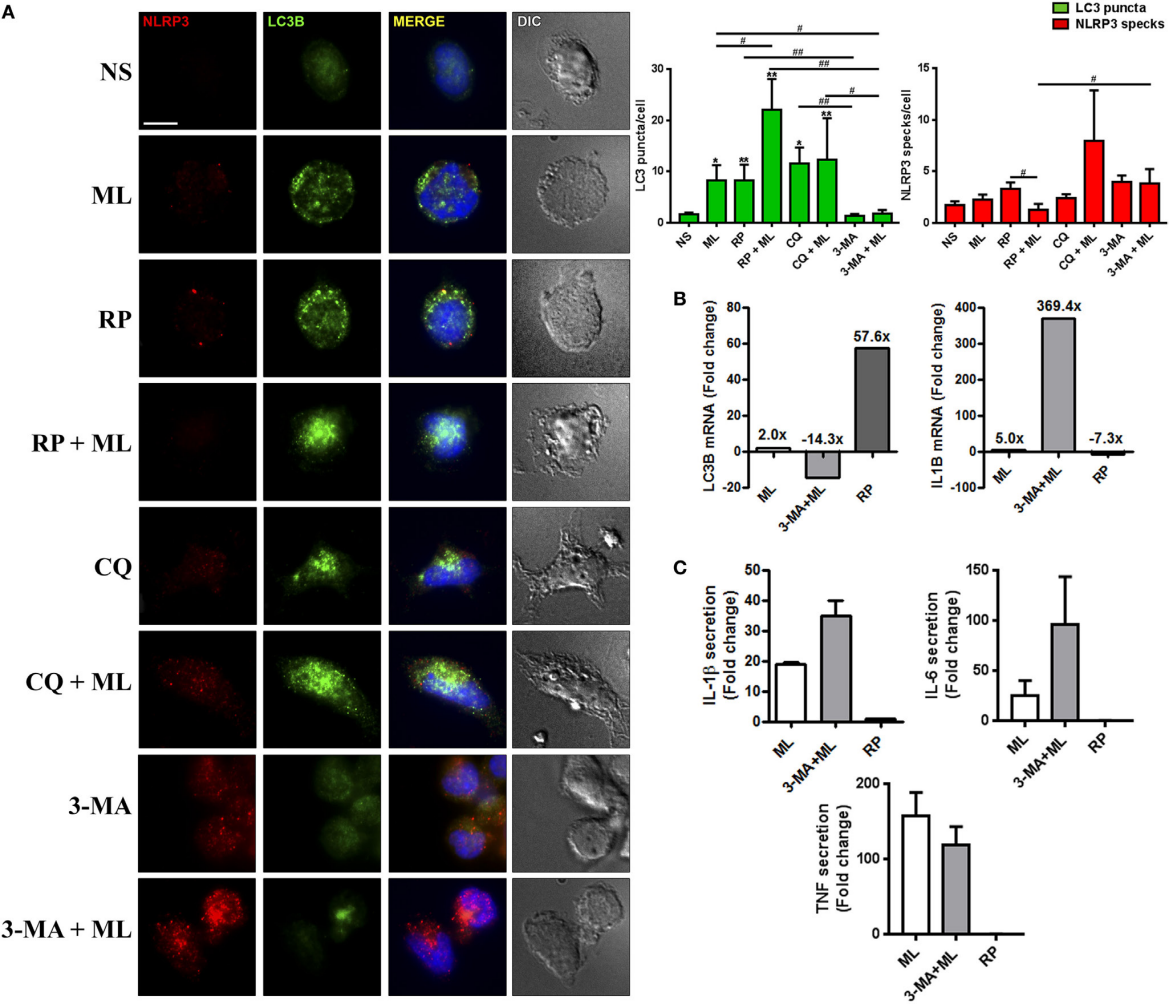
Autophagic pathway regulates inflammasome activation in blood-derived monocytes stimulated with *M. leprae*. **(A–C)** Monocytes were purified from healthy donors’ peripheral blood mononuclear cells by 2-h adherence and stimulated with 10 µg/mL *M. leprae* (ML) in the presence or absence of autophagy regulators for 18 h. Autophagy was triggered with 200 ng/mL rapamycin (RP), and inhibited with 100 µM chloroquine (CQ, autophagic flux blocker) and 10 µM 3-methyladenine (3-MA). **(A)** Monocytes stimulated with *M. leprae*, RP, CQ, and 3-MA were fixed and stained with anti-NLRP3 (red), anti-LC3B (green) antibodies and DAPI (blue). Monocytes stimulated with *M. leprae* increase the number of LC3-II-decorated autophagosomes (puncta) per cell, which is reforced by RP addition, and reverted by 3-MA. Autophagy blocking with CQ and 3-MA was able to increase NLRP3 specks numbers per cell in relation to the well stimulated with *M. leprae* and RP. Immunofluorescence images were quantified and bars represent the mean values of the number of LC3 puncta and NLRP3 specks per cell ± SEM (*n* = 3). *(in relation to NS) or ^#^(indicated by the dashes) *p* < 0.05. Scale bar: 10 µm. **(B)** Purified mRNAs from monocyte cultures stimulated with *M. leprae*, the autophagy blocker 3-MA, and RP, as a control of autophagy activation, were analyzed by RT-qPCR for *LC3B* and *IL1B*. Bars represent the mean of the fold change values in relation to the non-stimulated controls of three independent experiments with similar results. **(C)** The IL-1β, IL-6, and TNF levels in the monocyte cultures supernatants were assessed by ELISA. Bars represent the fold change values in relation to the non-stimulated controls ± SEM (*n* = 3).

Immunofluorescence analysis revealed that induction of autophagy by dead *M. leprae* or RP treatment was able to increase LC3 puncta formation in monocytes (Figure 5A). Exposure to these stimuli also triggered a degradation of inflammasomes, as observed by the reduced numbers of NLRP3 specks, similar to levels of unstimulated cells (Figure 5A). Autophagy was sensitive to inhibition by 3-MA leading to blockage of the autophagosome generation, as well as to the inhibitor of autophagosome-lysosome fusion CQ, which led to accumulation of LC3-positive dots (Figure 5A). However, we found that the NLRP3 activation was insensitive to neutralization of autophagy by CQ, since only 3-MA treatment promoted the increase of NLRP3 specks, although there is no significant difference in the number of NLRP3 specks by themselves in comparison with control cells, which was already expected since in these cells there is not much stimulus (e.g., first signal) for inflamassome activation (Figure 5A).

By treating *M. leprae*-stimulated monocytes with RP, which trigger autophagy by inhibiting the nutrient and energy sensor mTOR, we showed an even higher increase in the number of LC3 puncta per cell with reduced amounts of NLRP3 specks (Figure 5A). In contrast, addition of CQ or 3-MA together with *M. leprae* was able to increase the number of NLRP3 specks observed per cell, suggesting an increase in inflammasome activation, which was followed by autophagy deactivation at the same time, as seen through the increase or decrease of LC3-positive vesicles in CQ- or 3-MA-treated *M. leprae*-stimulated monocytes, respectively (Figure 5A).

In order to confirm this observation, the mRNA expression of *LC3B* and *IL1B* was evaluated in blood-derived monocytes cultures stimulated with γ-irradiated *M. leprae* and the autophagy blocker 3-MA. RP, used as a positive control for autophagy activation, was able to induce 57.6-fold increase in *LC3B* expression as compared to the non-stimulated control (Figure 5B). The *M. leprae* stimuli was able to increase in two-fold the gene expression of *LC3B* (Figure 5B). This increase was reverted 16.3-fold by the treatment with 3-MA (−14.3-fold in relation to non-stimulated) (Figure 5B). Conversely, autophagy induction was able to decrease in −7.3-fold the transcription of *IL1B* in comparison with non-stimulated monocytes (Figure 5B), and the inhibition of autophagy with 3-MA was able to increase the *IL1B* gene expression in 364.4-fold in relation to *M. leprae*-stimulated monocytes (369.4-fold change in relation to non-stimulated monocytes) (Figure 5B).

Next, we evaluated the secretion of IL-1β, and the pro-inflammatory cytokines IL-6 and TNF in the *M. leprae* and 3-MA-stimulated monocyte cultures supernatants. The stimuli with *M. leprae* was able to increase the production of IL-1β and the other pro-inflammatory cytokines evaluated in comparison to non-stimulated cultures (Figure 5C). The autophagy blockade by 3-MA treatment in conjunct with *M. leprae* was able to increase the secretion of IL-1β and IL-6, but not TNF as compared to the wells treated just with the mycobacteria (Figure 5C).

Together, these data indicate that autophagy is an important regulator of inflammasome activation during *M. leprae* infection and helps to control the exacerbated inflammation that leads to reversal reaction episodes onset.

## Discussion

The identification of biomarkers of leprosy reaction is an urgent demand. Leprosy reactions are responsible for nerve lesions and physical incapacities caused by leprosy (1). Reversal reaction may occur in borderline patients and also in subpolar lepromatous patients. It is described as an exacerbation of pre-occurring lesions in the skin and nerves (16, 32). Several studies have described possible biomarkers of reverse reaction (16, 18, 19, 33–36).

The outcome of reversal reaction is attributed to a sudden shift of the immune system, with an increase in the cell-mediated response against the bacilli (37). In the present study, we recruited multibacillary patients at the diagnosis and realized a 24-month follow-up to classify them accordingly the occurrence or not of reversal reaction. Leprosy reactions may occur in any time, before, during, and even after completing the multidrug therapy. One limitation of our study was short period follow-up, of only 1 year after the end of treatment. Besides that, our strategy was sufficient to identify the involvement of autophagy impairment and inflammasome activation as the mechanisms responsible for reversal reaction outcome in multibacillary patients.

Cellular immune mechanisms are associated with the development of reversal reaction. To identify the immune pathways related to reversal reaction we evaluated the expression of a total of 88 innate and adaptative immune genes in skin lesions from multibacillary patients. Analysis of innate and adaptive immune pathways demonstrated that *TLR*2 expression was significantly different when comparing the group that did not develop reaction during treatment (WR) and the group that develop reversal reaction (T1R). Although TLR2 expression was significantly increased in cells from skin lesions, analysis of *M. leprae*-stimulated monocytes did not shown differences between cells from WR and T1R groups (not shown), suggesting that there is a modulation of local immune response. A previous study has described *TLR2* polymorphisms associated with reversal reaction (38) and analysis of skin cells from patients with reversal reaction demonstrated that corticosteroids may reduce both gene and protein expression of TLR2 (39). Our present study demonstrated that in patients that develop reversal reaction in the future (T1R), *TLR2* is modulated differentially even before the appearance of the clinical symptoms of the reactional episode.

Several studies have demonstrated that TLR2 is associated with autophagy (40–43). However, more recently, it was demonstrated that both microRNAs miR0125a and miR-23a-5p inhibit autophagy activation during *M. tuberculosis* infection by mechanisms related to increased TLR2 expression (40, 44). Future studies will determine if the increased TLR2 expression in T1R group is related to the activation of pro-inflammatory signals associated to the initial stages of the development of reversal reaction or if it is contributing for the blockade of autophagy in multibacillary group.

Gene-expression analysis demonstrated an upregulation of autophagic genes in WR group when compared with T1R. PCR array analysis demonstrated that those genes which had their expressions significantly upregulated in WR group are associated with fibrosis (*FRS3*), vesicle trafficking (*SEC24C*), cellular viability (*LETM1*), protection, maintenance and adhesion of lysosomes (*LAMP2*), neuronal migration and neurite branching and elongation (*ULK4*), folding and assembly of proteins in the endoplasmic reticulum (*HSPA5*), and regulation of inflammation (*GPSM3*). In addition, *APOL1*, a gene that codifies a protein that is part of IFN-γ inducible host defense against *M. leprae*, is upregulated in WR group (45). It is possible that mechanisms related to basal autophagy may be important to sustain *M. leprae* infection in host cells, since autophagy is observed in patients in distinct regulation pattern in the different clinical forms of the disease, being downregulated in cells from multibacillary patients that have predisposition to develop reversal reaction.

*MLST8* was the unique gene significantly upregulated in T1R group. mLST8 is a subunit of both mTORC1 and mTORC2 being necessary for the mTOR kinase activation (46). The increase of mLST8 could partially explain the autophagy impairment in T1R since autophagy is negatively regulated by mTOR (47, 48).

Previous study comparing gene-expression pattern in patients with the different polar forms of leprosy demonstrated an increase in type I IFN in lepromatous patients (49) and that type 1 IFN may negatively regulate the responses of type II IFN (49). More recently, our group demonstrated that the gene encoding 2′-5′ oligoadenylate synthetase-like is upregulated in *M. leprae*-infected human macrophage cell lineages, primary monocytes, and skin lesion specimens from patients with lepromatous leprosy (50). Type I IFNs is important for the suppression of inflammasome and it was reported to inhibit NLRP3-activated inflammasome *via* STAT1 (51, 52). In the present study, the downregulation of autophagy pathways in cells from T1R group was accompanied by the increase in NLRP3 inflammasome-IL-1β pathway.

Inflammasomes are multiprotein oligomers that controls the maturation of IL-1β-related cytokines through activation of caspase-1 (53, 54). Pro-inflammatory cytokines were previously associated with reversal reaction occurrence, including IL-1β (17, 55–57) but mechanisms of the reactional episode outcome have not been elucidated. In the present work, it was not possible to determine if the bacilli or the inflammatory environment were responsible for mTOR activation in T1R group, however, the data presented here clearly suggest that the axis autophagy-inflammasome is crucial in the development or not of the reversal reaction in multibacillary patients. The strongest evidence of the involvement of inflammasome activation in the development of reversal reaction is the fact that in WR group *GPSM3* was overexpressed and a previous study demonstrated that GPSM3 is a negative regulator of IL-1β production triggered by NLRP3-dependent inflammasome activators (54), as well as the increase of the protein caspase-1 and secretion of IL-1β in the T1R group samples.

Wang et al. (58) demonstrated that upon inflammasome activation, inflammatory caspases cleave cGAS and render it inactive dampening of IFN activating pathways. It is possible that in the T1R group, the increase in inflammasome activation decreases type I IFN responses, contributing for the increase of type II IFN and other pro-inflammatory cytokines that could be associated with the reversal reaction onset.

*In vitro* experiments demonstrated that the blockade of autophagy pathway by 3-MA was able to increase NLRP3 expression in *M. leprae*-stimulated human monocytes, which was accompanied by an increase in *IL1B* mRNA. Previous studies have demonstrated that 3-MA leads to the accumulation of damaged-mitochondria-producing-ROS, and the activation of NLRP3 inflammasome, and consequently, causes IL-1β secretion (59). Analysis of cytokine production in *M. leprae*-stimulated cultures demonstrated that the blockade of autophagy by 3-MA did not affect the concentrations of TNF, but increased IL-1β and IL-6 secretion in *M. leprae*-stimulated cells. Previous studies have demonstrated that SNPs in *IL6* are associated with the outcome of reactional episodes (60) and IL-6 has been determined as a plasma marker for type 1 reaction (16).

In conclusion, the overall results of the present work demonstrated that in multibacillary patients more prone to develop reversal reaction there is an overexpression of both TLR2 and NLRP3 inflammasome-IL-1β pathway that is consequence of downregulation of autophagy. The data suggest that inappropriate inflammasome activation may contribute for the development of reversal reaction and open the perspective for the use of pro-autophagic drugs in the control not only of the bacillary load, as previously shown (7), but also the outcome of reversal reaction in multibacillary patients.

## Ethics Statement

This study was carried out in accordance with the recommendations of the Human Ethics Committee of the Oswaldo Cruz Foundation (approved protocol CAAE 34239814.7.0000.5248). All subjects gave their written informed consent in accordance with the Declaration of Helsinki.

## Author Contributions

MGMB, and ROP designed research and wrote the original draft; MGMB, BJAS, TQA, RBSP, HF, PRA, JAPO, and JACN performed research; MGMB, BJAS, GMSS, and ROP analyzed and interpreted the data; MGMB, BJAS, ENS, and ROP edited and reviewed the manuscript.

## Conflict of Interest Statement

The authors declare that the research was conducted in the absence of any commercial or financial relationships that could be construed as a potential conflict of interest.
