# Supplementary figures and images for: Autophagy Impairment Is Associated With Increased Inflammasome Activation and Reversal Reaction Development in Multibacillary Leprosy
Source: Front Immunol. 2018 Jun 4;9:1223. doi: 10.3389/fimmu.2018.01223 (PMC5994478; doi:10.3389/fimmu.2018.01223)

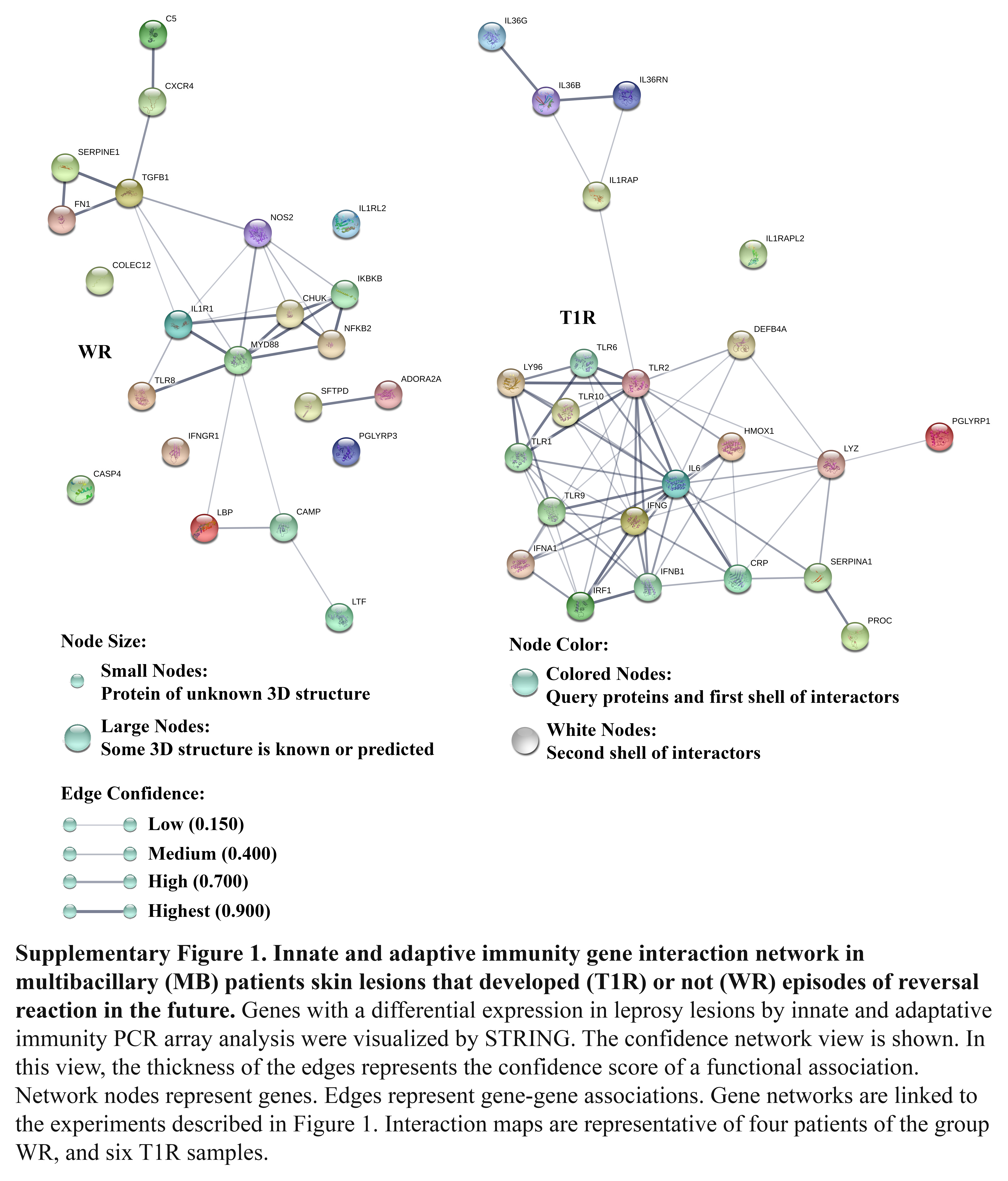

Supplement: Supplementary file 1 [file image_1.TIF]

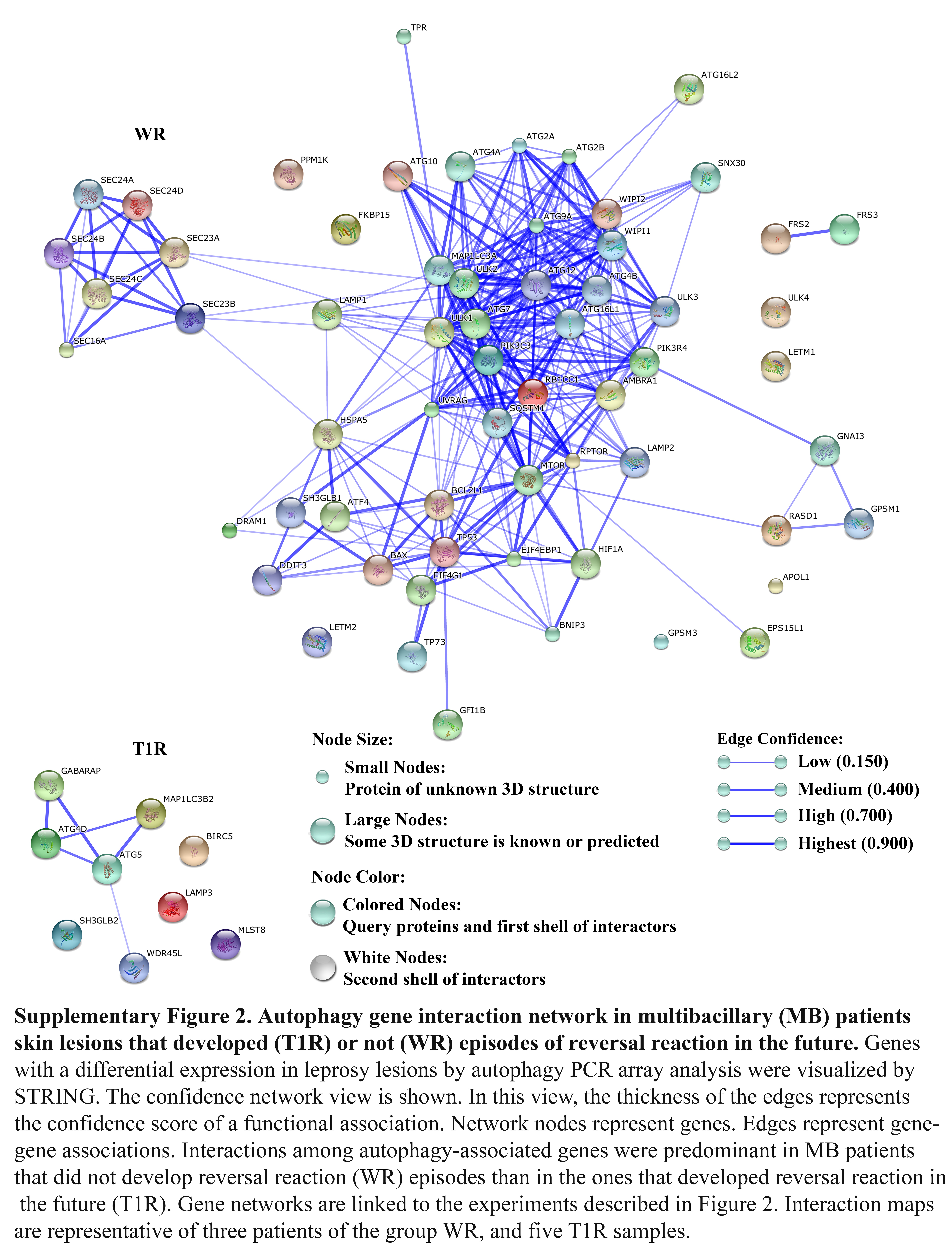

Supplement: Supplementary file 2 [file image_2.JPEG]
